# Dextroamphetamine (but Not Atomoxetine) Induces Reanimation from General Anesthesia: Implications for the Roles of Dopamine and Norepinephrine in Active Emergence

**DOI:** 10.1371/journal.pone.0131914

**Published:** 2015-07-06

**Authors:** Jonathan D. Kenny, Norman E. Taylor, Emery N. Brown, Ken Solt

**Affiliations:** 1 Department of Anesthesia, Critical Care, and Pain Medicine, Massachusetts General Hospital, Boston, Massachusetts, United States of America; 2 Department of Anaesthesia, Harvard Medical School, Boston, Massachusetts, United States of America; 3 Institute for Medical Engineering and Science, Massachusetts Institute of Technology, Cambridge, Massachusetts, United States of America; 4 Department of Brain and Cognitive Sciences, Massachusetts Institute of Technology, Cambridge, Massachusetts, United States of America; University of Oxford, UNITED KINGDOM

## Abstract

Methylphenidate induces reanimation (active emergence) from general anesthesia in rodents, and recent evidence suggests that dopaminergic neurotransmission is important in producing this effect. Dextroamphetamine causes the direct release of dopamine and norepinephrine, whereas atomoxetine is a selective reuptake inhibitor for norepinephrine. Like methylphenidate, both drugs are prescribed to treat Attention Deficit Hyperactivity Disorder. In this study, we tested the efficacy of dextroamphetamine and atomoxetine for inducing reanimation from general anesthesia in rats. Emergence from general anesthesia was defined by return of righting. During continuous sevoflurane anesthesia, dextroamphetamine dose-dependently induced behavioral arousal and restored righting, but atomoxetine did not (n = 6 each). When the D1 dopamine receptor antagonist SCH-23390 was administered prior to dextroamphetamine under the same conditions, righting was not restored (n = 6). After a single dose of propofol (8 mg/kg IV), the mean emergence times for rats that received normal saline (vehicle) and dextroamphetamine (1 mg/kg IV) were 641 sec and 404 sec, respectively (n = 8 each). The difference was statistically significant. Although atomoxetine reduced mean emergence time to 566 sec (n = 8), this decrease was not statistically significant. Spectral analysis of electroencephalogram recordings revealed that dextroamphetamine and atomoxetine both induced a shift in peak power from δ (0.1–4 Hz) to θ (4–8 Hz) during continuous sevoflurane general anesthesia, which was not observed when animals were pre-treated with SCH-23390. In summary, dextroamphetamine induces reanimation from general anesthesia in rodents, but atomoxetine does not induce an arousal response under the same experimental conditions. This supports the hypothesis that dopaminergic stimulation during general anesthesia produces a robust behavioral arousal response. In contrast, selective noradrenergic stimulation causes significant neurophysiological changes, but does not promote behavioral arousal during general anesthesia. We hypothesize that dextroamphetamine is more likely than atomoxetine to be clinically useful for restoring consciousness in anesthetized patients, mainly due to its stimulation of dopaminergic neurotransmission.

## Introduction

Patients recovering from general anesthesia can present significant clinical challenges including airway and oxygenation problems [1], emergence delirium [2, 3], cognitive dysfunction [4, 5], and delayed emergence [6]. Limited tools are available to treat these problems because the mechanisms underlying anesthetic emergence have not been elucidated. However, recent evidence suggests that arousal circuits projecting from nuclei in the brainstem and the midbrain are important for the recovery of consciousness after general anesthesia [7]. At the level of neural circuits, arousal-promoting cholinergic [8, 9], monoaminergic [10–13] and orexinergic [14–16] neurons have been implicated in anesthetic emergence, but their specific roles in restoring consciousness and cognitive function remain unclear.

In clinical practice, emergence from general anesthesia is elicited merely by discontinuing the general anesthetic, and it is treated as a passive process governed by the pharmacokinetics of anesthetic drug elimination. In contrast, methylphenidate induces active emergence, or reanimation, from general anesthesia in rodents [13, 17]. Methylphenidate inhibits reuptake transporters for dopamine and norepinephrine in the brain [18], and both neurotransmitters are known to promote arousal [19]. More recently, intravenous administration of a D1 dopamine receptor agonist [20] as well as electrical stimulation of the ventral tegmental area (VTA) [21] have been shown to induce reanimation in anesthetized rodents, suggesting that dopamine release by VTA neurons causes a profound arousal response sufficient to reverse the behavioral effects of general anesthesia. However, less is known about the role of norepinephrine in reanimation.

Like methylphenidate, dextroamphetamine and atomoxetine are prescribed for the treatment of Attention Deficit Hyperactivity Disorder (ADHD), but each drug has a distinct mechanism of action. Dextroamphetamine triggers the direct release of dopamine and norepinephrine in the brain, whereas atomoxetine is a reuptake inhibitor that is highly selective for the norepinephrine transporter (NET) over the dopamine transporter (DAT) [22]. In this study, we tested the hypothesis that dextroamphetamine and atomoxetine induce reanimation from general anesthesia in rats, and used these two drugs to probe the relative importance of dopamine and norepinephrine in reanimation. In addition, we recorded the electroencephalogram (EEG) during continuous sevoflurane anesthesia and analyzed the spectral changes induced by dextroamphetamine and atomoxetine. Finally, during continuous sevoflurane anesthesia, rats were administered SCH-23390 prior to dextroamphetamine and atomoxetine. Although it is commonly regarded as a selective D1 dopamine receptor antagonist, SCH-23390 is also known to inhibit α adrenergic receptors [23], so this drug was used to further test the roles of dopamine and norepinephrine in reanimation.

## Materials and Methods

### Ethics Statement

All studies were approved by the Massachusetts General Hospital Institutional Animal Care and Use Committee (Permit number: A3596-01), and were carried out in strict accordance with recommendations in the Guide for the Care and Use of Laboratory Animals of the National Institutes of Health. Invasive procedures and experiments were always performed under general anesthesia with isoflurane, sevoflurane or propofol, and appropriate analgesia was provided after surgery. All efforts were made to minimize animal suffering.

### Animal Care and Use

Seventeen adult male Sprague-Dawley rats (Charles River Laboratories, Wilmington, MA) were used for this study. Fourteen were used for the behavioral experiments under general anesthesia with sevoflurane (n = 6) and propofol (n = 8). Three additional rats with pre-implanted extradural electrodes were used for electroencephalogram (EEG) experiments. Different doses of dextroamphetamine or atomoxetine were administered on different days in random order. Only one experiment was conducted on each animal on any given day, and at least three days of rest were provided between experiments. Animals were kept on a standard day-night cycle (lights on at 7:00 AM and off at 7:00 PM), and all experiments were performed during the day.

### Preparation and Delivery of Drugs

Dextroamphetamine hemisulfate, atomoxetine hydrochloride, and SCH-23390 (R(+)-7-Chloro-8-hydroxy-3-methyl-1-phenyl-2,3,4,5-tetrahydro-1H-3-benzazepine hydrochloride) were purchased from Sigma-Aldrich (St. Louis, MO). Propofol was obtained from APP Pharmaceuticals (Shaumburg, IL). All drugs (except for the inhaled anesthetic sevoflurane) were administered intravenously via a lateral tail vein catheter. Drug solutions were freshly prepared each day by weighing and dissolving the drug in 1.0 ml of sterile normal saline before administration. The intravenous catheter and attached tubing (approximate total volume 2.0 ml) were always flushed with 3.0 ml of normal saline after drug administration to ensure complete drug delivery.

### Administration of Intravenous Agents During Continuous Sevoflurane Anesthesia

In studies of anesthetic emergence, passive emergence (i.e. discontinuing the general anesthetic and determining time to righting) is commonly used to assess changes in arousal state. For inhaled anesthetics, however, the pharmacokinetics of anesthetic clearance can be greatly affected by changes in respiration [13]. Therefore in order to test specifically for changes in arousal induced by dextroamphetamine and atomoxetine, all experiments with sevoflurane were performed during continuous inhalation of a fixed dose under steady-state conditions. With this method, any changes in respiratory drive induced by dextroamphetamine or atomoxetine could not account for changes in arousal, since the anesthetic dose remained constant.

Rats were anesthetized with sevoflurane (4 to 6%) in oxygen and a 24-gauge intravenous catheter was placed in a lateral tail vein. A rectal temperature probe was inserted and the rat was placed in a custom built acrylic anesthetizing chamber with ports for anesthetic gas delivery, sampling, scavenging, and intravenous drug administration as previously described [13]. A heating pad was placed under the chamber to keep the core body temperature between 36.5°C and 37.5°C, and fresh gas flow was maintained at a minimum rate of two L/min. Anesthetic gas was continuously sampled from the distal portion of the cylindrical chamber using an Ohmeda 5250 anesthetic agent analyzer (GE Healthcare, Waukesha, WI).

To determine the minimum concentration of sevoflurane to maintain loss of righting, the inhaled concentration of sevoflurane (in oxygen) was initially held at 1.4%. If the rat exhibited any purposeful movement within 25 minutes such as lifting of the head, twisting of the torso, kicking, clawing, chewing, licking, or grooming, the concentration of sevoflurane was increased by 0.1%. At the final dose of sevoflurane, the rat remained supine and did not display any purposeful movements for 25 minutes, and this concentration was fixed for the remainder of the experiment. The intravenous catheter was then flushed with 2.0 ml of normal saline to ensure that the intravenous catheter was patent, and to confirm that injection of the vehicle did not elicit an arousal response. Five minutes after the saline injection, dextroamphetamine or atomoxetine was administered. Sevoflurane anesthesia was maintained at the same dose for ten additional minutes or until righting was restored, whichever came first. To establish a dose-response relationship, three different doses of dextroamphetamine and atomoxetine (0.3 mg/kg, 1 mg/kg, and 3 mg/kg) were administered on different days in random order.

In a separate experiment using the same animals, the same anesthetizing conditions were established with sevoflurane, but the D1 receptor antagonist SCH-23390 (0.2 mg/kg IV) was administered instead of the normal saline control, followed five minutes later by dextroamphetamine (1 mg/kg IV). After intravenous drug administration each animal continued to inhale the same dose of sevoflurane for ten minutes or until restoration of righting occurred, whichever came first.

### Time to Emergence after a Propofol Bolus

For intravenous general anesthetics such as propofol, drug elimination is primarily driven by hepatic metabolism and the process is unaffected by changes in respiration. Therefore, passive emergence after a single IV dose of propofol can be used to test for arousal changes induced by dextroamphetamine or atomoxetine, because changes in respiratory drive will not confound the results.

After placement of a lateral tail vein IV catheter under sevoflurane anesthesia, each rat was allowed to fully recover in room air until it regained the righting reflex and returned to a normal level of activity for at least ten minutes. A single dose of propofol sufficient to induce loss of righting (8 mg/kg IV) was then administered, and the animal was placed supine on a heating pad. Normal saline (vehicle), dextroamphetamine (1 mg/kg IV) or atomoxetine (1 mg/kg) was administered 45 seconds after the propofol injection. Time to emergence was defined as the time from propofol administration until restoration of righting (all four paws touching the ground). The same animals (n = 8) were used for the three experimental conditions (saline, dextroamphetamine, and atomoxetine) on different days in random order, with at least three days of rest between experiments.

### EEG Electrode Placement, Recording, and Spectral Analysis

In addition to the 14 rats used for behavioral experiments, three rats were used to record the EEG during sevoflurane general anesthesia. Extradural EEG electrodes were surgically implanted under isoflurane anesthesia as previously described [17]. Post-operative analgesia was provided for a minimum of 2 days following the procedure, and the animals were allowed to fully recover for a minimum of seven days before experiments were performed. The EEG signal was recorded using a QP511 Quad AC Amplifier System (Grass Instruments, West Warwick, RI) and a USB-6009 14-bit data acquisition board (National Instruments, Austin, TX). The sampling rate was 500 Hz, and a notch filter was used to eliminate 60 Hz noise.

After placement of a tail vein IV catheter and rectal temperature probe under sevoflurane anesthesia, rats were placed in the anesthetizing chamber in the prone position and the inhaled dose of sevoflurane was fixed at a concentration (2.0 to 2.3%) that produced a δ-dominant (0.1–4 Hz) EEG pattern. The change in body position and the slightly higher anesthetic dose were chosen to minimize motion artifacts after the administration of arousal-promoting agents, as previously described [13]. After 35 minutes of continuous sevoflurane exposure to ensure steady-state conditions, normal saline was administered intravenously. Five minutes later, dextroamphetamine (1 mg/kg IV) or atomoxetine (3 mg/kg IV) was administered while sevoflurane anesthesia was maintained at the same dose for 20 additional minutes. In separate experiments, the D1 dopamine receptor antagonist SCH-23390 was administered five minutes before dextroamphetamine or atomoxetine.

Spectral analysis was performed using MATLAB R2015a (Mathworks, Natick, MA) and the Chronux software package (Cold Spring Harbor, NY) [24]. Spectrograms were computed within the frequency range of 0.1 to 35 Hz using a two-second sliding window stepped by 0.1 seconds. The half-bandwidth of the spectrogram was 1 Hz, with 3 tapers used in its construction.

### Statistical Analysis of the Effects of Dextroamphetamine and Atomoxetine on Emergence Times, Return of Righting Responses, and Power Spectral Densities

MATLAB R2015a was used for statistical analysis and, when possible, results are reported in terms of 95% confidence intervals based on a percentile bootstrap procedure.

Time to righting was compared among the saline, dextroamphetamine, and atomoxetine groups using 95% confidence intervals. Confidence intervals were constructed around the mean using the percentile bootstrap [25]. The confidence intervals around the difference of means between two groups were used to test for statistical significance. If the 95% confidence bounds around the difference of means were both positive, then there was a significant increase. If the 95% confidence bounds around the difference of means were both negative, then there was a significant decrease. If the lower 95% confidence bound was negative and the upper 95% confidence bound was positive, then there was no significant difference.

A Bayesian Monte Carlo procedure was used to compute 95% credible intervals to assess the effects of dextroamphetamine and atomoxetine on return of righting during continuous sevoflurane general anesthesia, as described previously [13, 17, 20]. Instead of p-values, for the Bayesian analyses, we computed the posterior probability that the propensity to right in one group was greater than in another. The result was considered significant if the posterior probability was greater than 0.95.

Group power spectral densities were computed across animals using two minutes of EEG data before drug administration, and two minutes of EEG data after drug administration. Power spectral densities were calculated using two-second bins from 0.1 to 35 Hz within each window, yielding 60 estimates for every two minutes of EEG data. Power spectral densities were then pooled across all three rats, giving 180 power spectral estimates before drug administration, and 180 estimates after drug administration. Distributions of power spectral estimates were resampled to be independent and identically distributed using the time-frequency toggle [26]. Confidence intervals (95%) were calculated at each discrete frequency interval using the percentile bootstrap.

## Results

### Dextroamphetamine Induces Reanimation during Continuous Sevoflurane General Anesthesia

**Fig 1:**
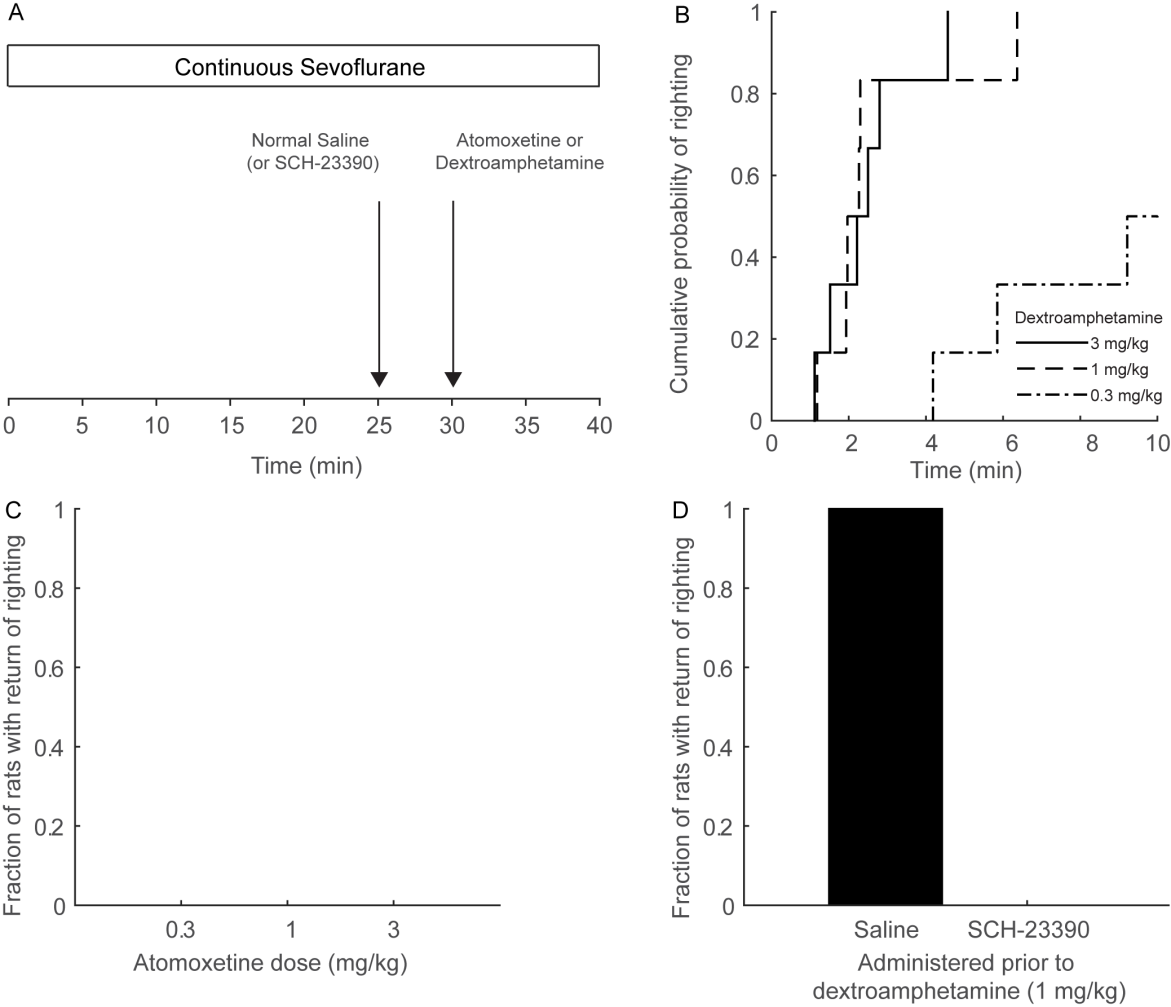
Dextroamphetamine restores righting during continuous sevoflurane general anesthesia, but atomoxetine does not. (A) Rats inhaled sevoflurane at a dose sufficient to maintain loss of righting for at least 25 consecutive minutes. They then received intravenous normal saline or SCH-23390 (0.2 mg/kg). Five minutes later, atomoxetine or dextroamphetamine was administered intravenously. Sevoflurane was maintained at the same dose for ten additional minutes, or until return of righting occurred. (B) Empirical cumulative distribution function for time to righting after the administration of dextroamphetamine. Dextroamphetamine restored righting within 10 minutes in 6/6 rats at doses of 1 mg/kg and 3 mg/kg IV, and in 3/6 rats at a dose of 0.3 mg/kg IV. (C) Atomoxetine did not restore righting within ten minutes of administration in any animals, regardless of the dose (n = 6 each). Even at the highest dose (3 mg/kg IV) atomoxetine did not elicit any behavioral signs of arousal. (D) In rats pretreated with SCH-23390, dextroamphetamine (1 mg/kg IV) did not restore righting in any animals (n = 6).

Fig 1A illustrates the protocol for this experiment. After establishing an inhaled concentration of sevoflurane sufficient to maintain loss of righting for 25 minutes, normal saline was administered intravenously. No animals exhibited a behavioral arousal response after the injection of normal saline. Five minutes later, atomoxetine or dextroamphetamine was administered. The final mean inhaled concentration of sevoflurane was 1.7% (95% CI: 1.6 to 1.7%) for both the atomoxetine and dextroamphetamine groups.

During continuous sevoflurane anesthesia, dextroamphetamine induced a vigorous arousal response (e.g. lifting of the head, twisting of the torso, kicking, clawing, chewing, licking, and/or grooming) and restored the righting reflex in a dose-dependent fashion (Fig 1B), despite continuous sevoflurane general anesthesia. At a dose of 0.3 mg/kg, dextroamphetamine restored the righting reflex within 10 minutes in 3/6 rats. When 1 mg/kg or 3 mg/kg was administered, dextroamphetamine restored righting in 6/6 rats. The mean time to righting after 1 mg/kg was 160 sec (95% CI: 101 to 253 sec, n = 6) and after 3 mg/kg it was 147 sec (95% CI: 100 to 204 sec, n = 6), but this difference was not statistically significant (mean difference: 13 sec; 95% CI: -74 to 117 sec) suggesting that maximum efficacy was achieved at a dextroamphetamine dose of 1 mg/kg. The Bayesian 95% CIs for the difference in propensities to have restoration of righting was -0.03 to 0.74 for saline versus 0.3 mg/kg dextroamphetamine, 0.38 to 0.97 for saline versus 1 mg/kg dextroamphetamine, and 0.38 to 0.97 for saline versus 3 mg/kg dextroamphetamine. The posterior probability that the difference was greater than zero was greater than 0.95 for all comparisons, indicating that the results were statistically significant.

However, under the same experimental conditions atomoxetine did not restore righting in any animals (n = 6) during continuous sevoflurane general anesthesia, even at the highest dose of 3 mg/kg (Fig 1C). Similar to normal saline, arousal behaviors were not observed after the administration of atomoxetine during sevoflurane anesthesia.

### SCH-23390 Inhibits Dextroamphetamine-induced Return of Righting during Continuous Sevoflurane General Anesthesia

In separate experiments, SCH-23390 (0.2 mg/kg IV) was administered instead of normal saline during continuous sevoflurane general anesthesia. Five minutes later, dextroamphetamine (1 mg/kg IV) was administered. Although 4/6 animals exhibited some signs of behavioral arousal such as eye opening and lifting of the head, none had return of righting within 10 minutes of dextroamphetamine administration. The Bayesian 95% CI for rats that received SCH-23390 versus normal saline was 0.37 to 0.97. The posterior probability was greater than 0.95, indicating that the results were statistically significant.

### Dextroamphetamine Decreases Time to Emergence from Propofol General Anesthesia

**Fig 2:**
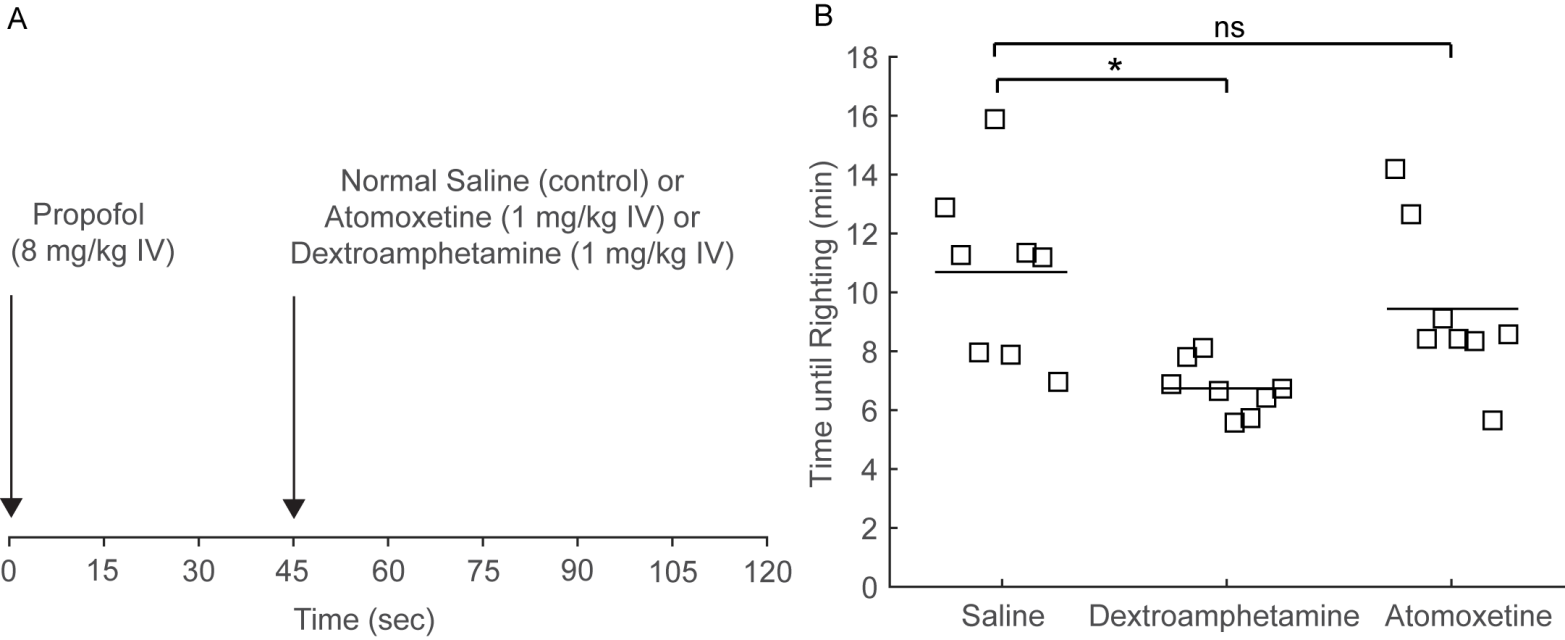
Dextroamphetamine significantly decreases time to emergence from propofol anesthesia, but atomoxetine does not. (A) Rats received a bolus of propofol (8 mg/kg IV), followed by normal saline (vehicle), atomoxetine (1 mg/kg IV) or dextroamphetamine (1 mg/kg IV). Time to emergence was defined as the time from administration of propofol to return of righting. (B) Scatter plot of time to emergence for rats that received normal saline, dextroamphetamine, and atomoxetine after propofol. The lines represent mean values. Dextroamphetamine caused a statistically significant decrease in time to emergence compared to normal saline, whereas atomoxetine did not.

Fig 2A shows the protocol for this experiment. Forty-five seconds after a single dose of propofol (8 mg/kg IV), normal saline (vehicle), dextroamphetamine (1 mg/kg IV) or atomoxetine (1 mg/kg IV) was administered, and time to emergence was recorded. Fig 2B shows a scatter plot of time to emergence after propofol for the normal saline, dextroamphetamine, and atomoxetine groups. The mean time to emergence for the normal saline group was 641 sec (95% CI: 530 to 759 sec, n = 8). In the dextroamphetamine group, mean time to emergence was 404 sec (95% CI: 371 to 439 sec, n = 8), and this reduction was statistically significant (mean difference: 237 sec; 95% CI: 121 to 358 sec). In the atomoxetine group, mean time to emergence was 566 sec (95% CI: 468 to 678 sec, n = 8) but this decrease in comparison to the normal saline group was not statistically significant (mean difference: 75 sec; 95% CI: -82 to 228 sec).

### Dextroamphetamine Induces EEG Changes during Continuous Sevoflurane General Anesthesia

**Fig 3:**
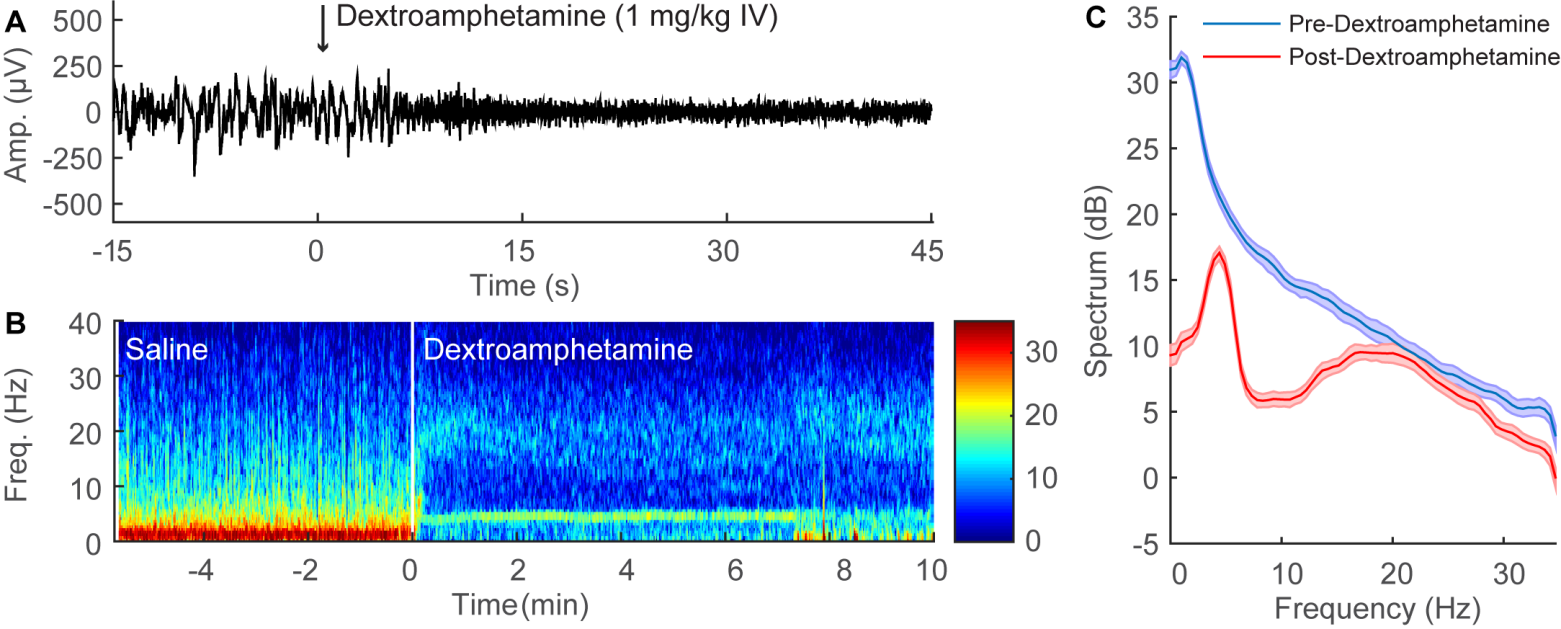
Dextroamphetamine administration induces significant EEG changes during continuous sevoflurane anesthesia. (A) Representative 60-second time-domain EEG recording from an individual rat during continuous sevoflurane anesthesia, with time zero indicating the administration of dextroamphetamine (1 mg/kg IV). The EEG pattern rapidly changes to a low amplitude, high frequency rhythm after the administration of dextroamphetamine. (B) Representative time-frequency domain spectrogram computed from 15 minutes of EEG data. Warm colors indicate high power at a given frequency, while cool colors indicate low power. A prompt shift in peak power from δ to θ is observed after the administration of dextroamphetamine. (C) Group power spectral density from all rats (n = 3), with shaded areas indicating 95% confidence intervals. Before dextroamphetamine administration (blue), δ power was dominant during continuous sevoflurane anesthesia. After dextroamphetamine administration (red), statistically significant reductions in power occurred at most frequencies below 15 Hz, and peak power was shifted to θ.

A representative time-domain EEG recording from an individual rat during continuous sevoflurane anesthesia is shown in Fig 3A. The administration of dextroamphetamine (1 mg/kg IV) decreased the amplitude and increased the frequency of the EEG waveform within 15 seconds. In order to analyze changes in spectral content over time, spectrograms were computed from the EEG data, and a representative result from an individual animal is shown in Fig 3B. During sevoflurane anesthesia, spectral power is concentrated in the lower δ frequency range (0.1–4Hz). However, the administration of dextroamphetamine (1 mg/kg IV) induced a rapid shift in peak power from δ to θ (4 to 8 Hz) frequencies.

Fig 3C shows group power spectral density from all three rats during continuous sevoflurane anesthesia, computed from two-minute periods before (blue) and after (red) the administration of dextroamphetamine (1 mg/kg IV). 95% confidence intervals around the mean power spectra were used to test whether dextroamphetamine induced statistically significant changes in the power spectrum. At any particular frequency, non-overlapping confidence intervals represent a statistically significant difference in power, whereas overlapping confidence intervals depict differences in power that are not statistically significant. Dextroamphetamine induced statistically significant decreases in power at most frequencies below 15 Hz, and shifted peak power from δ to θ.

### SCH-23390 Inhibits Dextroamphetamine-induced EEG Changes during Continuous Sevoflurane General Anesthesia

**Fig 4:**
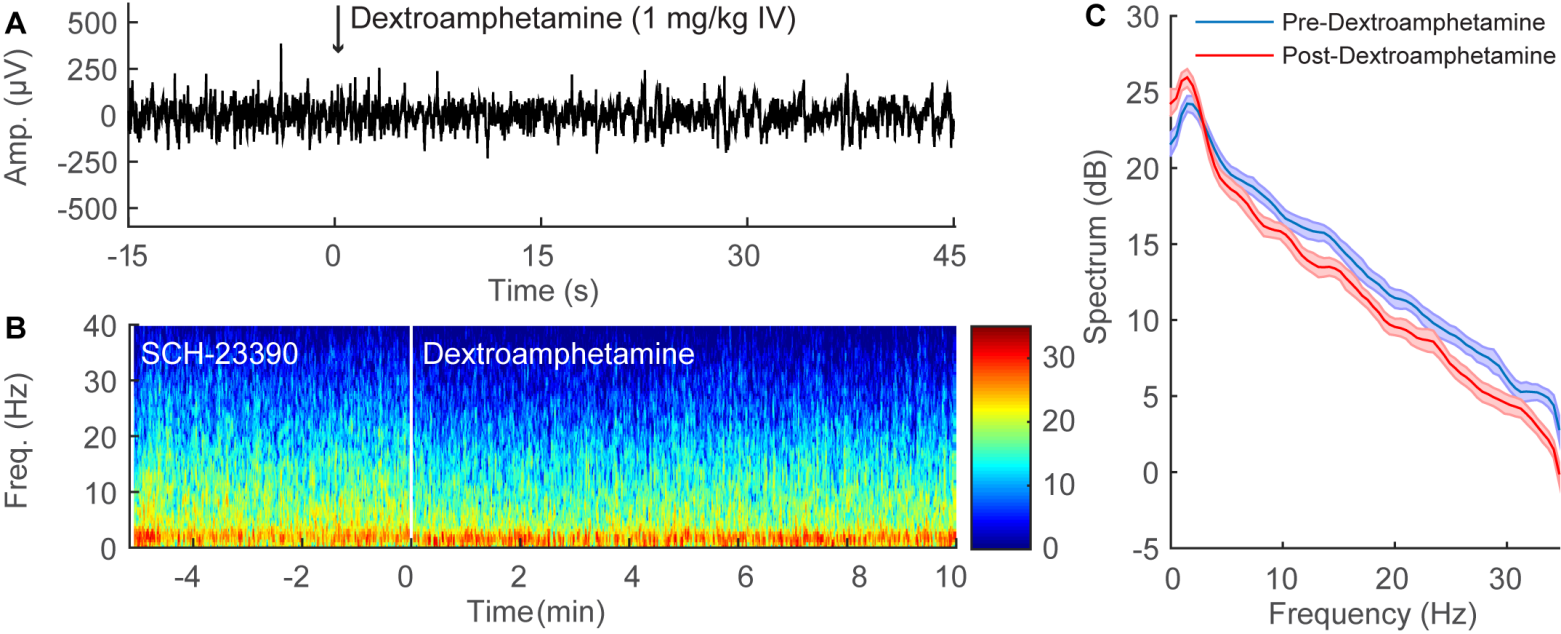
SCH-23390 inhibits the EEG changes induced by dextroamphetamine during continuous sevoflurane anesthesia. (A) Representative 60-second time-domain EEG recording from an individual rat during continuous sevoflurane anesthesia after pre-treatment with SCH-23390 (0.5 mg/kg IV), with time zero indicating the administration of dextroamphetamine (1 mg/kg IV). The EEG pattern remains essentially unchanged after dextroamphetamine administration. (B) Representative time-frequency domain spectrogram computed from 15 minutes of EEG data. After the administration of SCH-23390, dextroamphetamine does not induce a shift in peak power from δ to θ. (C) Group power spectral density from all rats (n = 3), with shaded areas indicating 95% confidence intervals. After the administration of SCH-23390 during continuous sevoflurane anesthesia (blue), dextroamphetamine administration (red) only induced small changes in power that were statistically significant.

A representative time-series EEG recording from an individual rat that received SCH-23390 (0.5 mg/kg IV) during continuous sevoflurane anesthesia is shown in Fig 4A. In contrast to the results obtained with normal saline pre-treatment (Fig 3A), dextroamphetamine (1 mg/kg IV) did not induce obvious EEG changes when administered 5 minutes after SCH-23390 (Fig 4A). A spectrogram computed from the EEG data (Fig 4B) shows that after the administration of SCH-23390 (0.5 mg/kg IV), dextroamphetamine (1 mg/kg IV) did not shift peak power from δ to θ. The group power spectral density from all three rats (Fig 4C) shows that after the administration of SCH-23390, dextroamphetamine only induced minor changes in the power spectrum that were statistically significant.

### Atomoxetine Induces EEG Changes Similar to Dextroamphetamine during Continuous Sevoflurane Anesthesia

**Fig 5:**
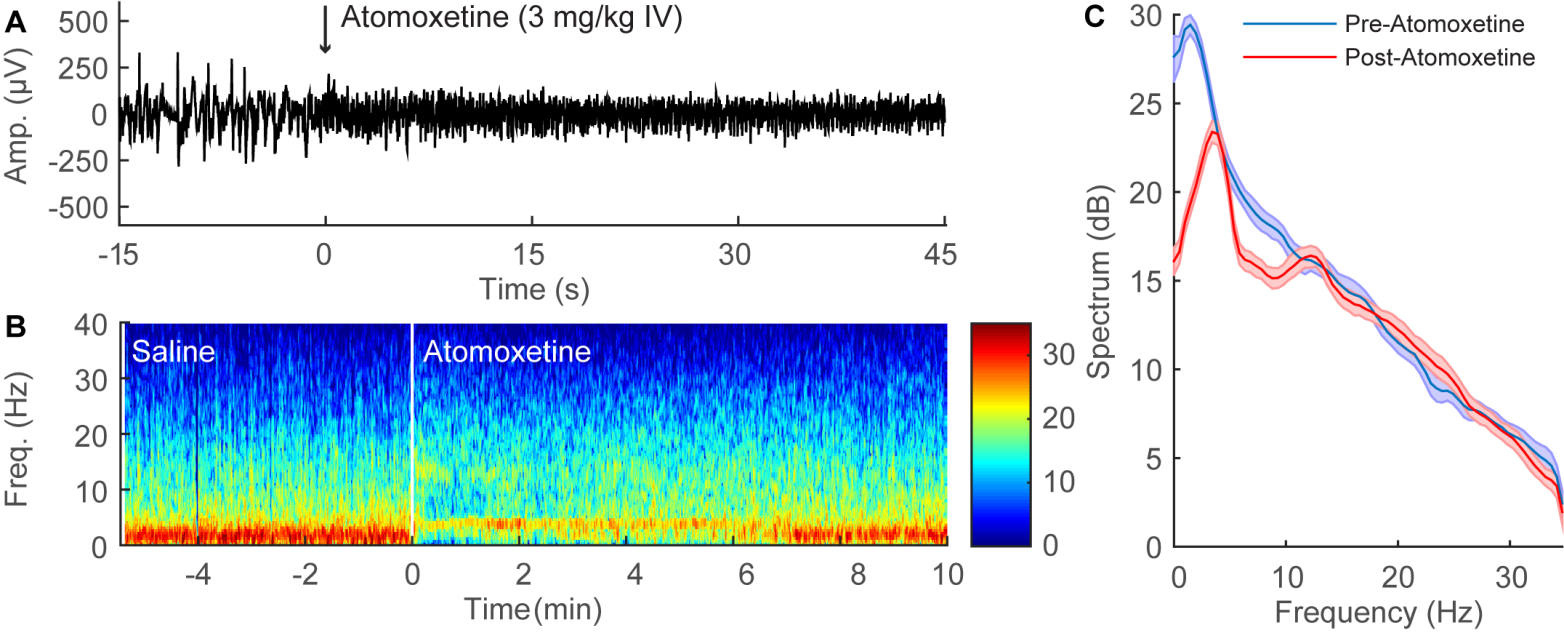
Atomoxetine administration induces significant EEG changes during continuous sevoflurane anesthesia. (A) Representative 60-second time-domain EEG recording from an individual rat during continuous sevoflurane anesthesia, with time zero indicating the administration of atomoxetine (3 mg/kg IV). The EEG pattern rapidly changes to a low amplitude, high frequency rhythm after the administration of atomoxetine. (B) Representative time-frequency domain spectrogram computed from 15 minutes of EEG data. A prompt shift in peak power from δ to θ is observed after the administration of atomoxetine. (C) Group power spectral density from all rats (n = 3), with shaded areas indicating 95% confidence intervals. Before atomoxetine administration (blue), δ power was dominant during continuous sevoflurane anesthesia. After atomoxetine administration (red), statistically significant reductions in power occurred at most frequencies below 10 Hz, and peak power was shifted to θ.

A representative time-series EEG recording from an individual rat during continuous sevoflurane anesthesia is shown in Fig 5A. Similar to dextroamphetamine, the administration of atomoxetine (3 mg/kg IV) rapidly decreased the amplitude and increased the frequency of the EEG waveform. A spectrogram computed from the EEG data (Fig 5B) shows that the administration of atomoxetine (3 mg/kg IV) induced an abrupt shift in peak power from δ to θ frequencies. The group power spectral density from all three rats (Fig 5C) shows that atomoxetine induced a statistically significant decrease in power at most frequencies below 10 Hz, and shifted peak power from δ to θ.

### SCH-23390 Inhibits Atomoxetine-induced EEG Changes during Continuous Sevoflurane Anesthesia

**Fig 6:**
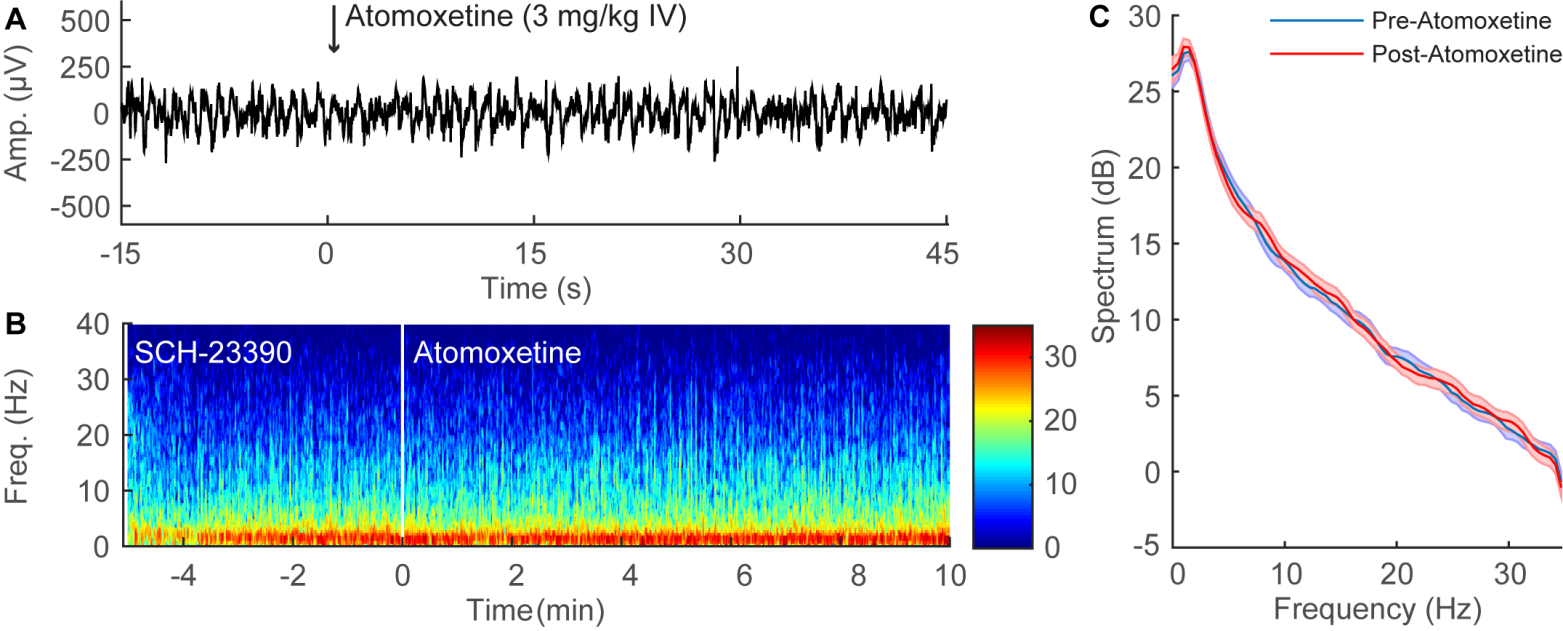
SCH-23390 inhibits the EEG changes induced by atomoxetine during continuous sevoflurane anesthesia. (A) Representative 60-second time-domain EEG recording from an individual rat during continuous sevoflurane anesthesia after pre-treatment with SCH-23390 (0.5 mg/kg IV), with time zero indicating the administration of atomoxetine (3 mg/kg IV). The EEG pattern remains essentially unchanged after atomoxetine administration. (B) Representative time-frequency domain spectrogram computed from 15 minutes of EEG data. After the administration of SCH-23390, atomoxetine does not induce a shift in peak power from δ to θ. (C) Group power spectral density from all rats (n = 3), with shaded areas indicating 95% confidence intervals. After the administration of SCH-23390 during continuous sevoflurane anesthesia (blue), atomoxetine administration (red) did not induce statistically significant changes in EEG power.

A representative time-series EEG recording from an individual rat that received SCH-23390 (0.5 mg/kg IV) during continuous sevoflurane anesthesia is shown in Fig 6A. In contrast to the results obtained with normal saline (Fig 5A), atomoxetine (3 mg/kg IV) did not induce obvious EEG changes when administered 5 minutes after SCH-23390 (Fig 6A). A spectrogram computed from the EEG data (Fig 6B) shows that after the administration of SCH-23390, atomoxetine did not shift peak power from δ to θ. The group power spectral density from all three rats (Fig 6C) shows that after the administration of SCH-23390, atomoxetine did not induce statistically significant changes in the power spectrum.

## Discussion

General anesthesia is a reversible, drug-induced state comprised of unconsciousness, amnesia, analgesia, and immobility (lack of movement in response to noxious stimulation) in the setting of hemodynamic stability [27]. Historically, the median alveolar concentration (MAC) of an inhaled anesthetic that produces immobility has been used to define anesthetic potency [28]. It was reported in the 1970s that amphetamine administration acutely increases MAC for halothane and cyclopropane in dogs [29–31], but unconsciousness is an anesthetic endpoint produced in the brain that is fundamentally distinct from immobility produced in the spinal cord [32–34], and little is known about the effects of amphetamine and other stimulant drugs on anesthetic-induced unconsciousness.

We previously reported that methylphenidate (an inhibitor of NET and DAT commonly used to treat ADHD) induces reanimation from general anesthesia with isoflurane [13] and propofol [17]. We also found that administration of a D1 dopamine receptor agonist [20] and electrical stimulation of the VTA [21] induce reanimation from general anesthesia, suggesting that VTA dopamine neurons play a key role. In this study we tested the hypotheses that two additional stimulant medications used to treat ADHD, dextroamphetamine and atomoxetine, also induce reanimation from general anesthesia. Dextroamphetamine causes the release of dopamine and norepinephrine from presynaptic terminals, whereas atomoxetine is a reuptake inhibitor that is approximately 1000 times more selective for NET than DAT [18]. The unique properties of these two agents allowed us to probe the relative contributions of dopaminergic and noradrenergic neurotransmission to reanimation from general anesthesia.

Because changes in respiratory function will alter the pharmacokinetic clearance of inhaled anesthetics and therefore affect time to emergence [13], all of our studies with sevoflurane were performed during continuous inhalation of a fixed dose sufficient to maintain loss of righting. Under these steady-state conditions respiratory changes cannot account for altered arousal, since the concentration of sevoflurane in the brain remains constant. Despite continuous sevoflurane anesthesia, intravenous dextroamphetamine rapidly induced arousal behaviors and restored righting in a dose-dependent manner. However, under identical experimental conditions, atomoxetine not only failed to restore righting, but also did not induce any observable behaviors consistent with an arousal response.

With the intravenous general anesthetic propofol, time to emergence after a single dose is an appropriate endpoint to test for changes in arousal, since respiratory changes have no effect on the liver metabolism of propofol. After receiving a standard dose of propofol (8 mg/kg IV), mean time to emergence was 37% faster for rats that received dextroamphetamine than rats that received the normal saline vehicle. This reduction in emergence time was statistically significant, and similar in magnitude to our previous results with methylphenidate [17]. However, under the same experimental conditions atomoxetine did not have a statistically significant effect on time to emergence from propofol anesthesia.

Because atomoxetine is highly selective for NET over DAT [18], the present results suggest that noradrenergic stimulation alone is insufficient to induce reanimation from general anesthesia. In contrast, dextroamphetamine, which promotes the release of dopamine and norepinephrine from presynaptic terminals, restored righting in a dose-dependent manner, similar to methylphenidate [13]. Although it is possible that higher doses of atomoxetine may have induced behavioral arousal, 3 mg/kg IV is among the highest doses reported in the literature for rats. Indeed, this dose of atomoxetine was high enough to cause significant EEG changes during general anesthesia in the present study, indicating that a sufficient quantity of the drug had reached the brain to induce neurophysiological changes. However, no behavioral arousal was observed.

Despite their contrasting behavioral effects during general anesthesia, atomoxetine and dextroamphetamine surprisingly produced similar neurophysiological findings, causing a shift in EEG peak power from δ to θ. These changes are similar to those induced by intravenous methylphenidate during isoflurane general anesthesia [13]. Because methylphenidate, dextroamphetamine and atomoxetine all enhance noradrenergic neurotransmission [18], the present results are consistent with the notion that noradrenergic stimulation during general anesthesia induces a shift in EEG peak power from δ to θ. However, in the case of atomoxetine this neurophysiological change does not correlate with a behavioral arousal response. Conversely, we previously reported that a D1 dopamine receptor agonist induces reanimation from isoflurane general anesthesia, but does not induce a θ-dominant rhythm on the EEG [20]. Therefore our results to date suggest that dopaminergic stimulation during general anesthesia induces a robust behavioral arousal response with relatively modest EEG changes, whereas noradrenergic stimulation induces a shift in EEG peak power from δ to θ without a concomitant behavioral arousal response.

SCH-23390 is widely used as a selective D1 dopamine receptor antagonist. In this study we found that pretreatment with SCH-23390 inhibited the arousal-promoting actions of dextroamphetamine during sevoflurane anesthesia, consistent with the notion that D1 receptors are primarily responsible for dextroamphetamine’s arousal-promoting effects [20]. However, SCH-23390 also inhibits α adrenergic receptors [23], which presumably explains why pretreatment with this drug during continuous sevoflurane anesthesia also inhibited the shift in EEG peak power from δ to θ induced by dextroamphetamine and atomoxetine.

It was recently reported that during isoflurane anesthesia, activation of noradrenergic neurons in the locus coeruleus (LC) using a Designer Receptor Exclusively Activated by Designer Drug (DREADD) approach shifted cortical EEG power from δ to θ [35]. This is consistent with our EEG findings using the NET inhibitor atomoxetine during sevoflurane anesthesia, suggesting that enhanced noradrenergic neurotransmission during general anesthesia shifts cortical EEG power from δ to 03B8. Although it was also reported that DREADD activation of LC noradrenergic neurons decreased time to emergence from isoflurane anesthesia [35], changes in respiratory drive can affect time to emergence when anesthetic administration is discontinued [13]. During continuous general anesthesia, when changes in respiratory drive cannot account for changes in behavioral arousal, we found that atomoxetine did not promote behavioral arousal or restore righting. This is in contrast to dextroamphetamine, methylphenidate [13], the D1 agonist Chloro-APB [20], and electrical stimulation of the VTA [21], all of which induced behavioral arousal and restored righting during continuous general anesthesia. Taken together, the available data suggest that dopaminergic stimulation induces a more robust behavioral arousal response than noradrenergic stimulation during general anesthesia.

Interestingly, it has been reported that atomoxetine increases not only extracellular norepinephrine levels, but also dopamine levels in the prefrontal cortex [36]. This is because extracellular dopamine is taken up by NET in the prefrontal cortex, due to a relative paucity of DAT [37]. However, this effect is apparently unique to the prefrontal cortex because unlike methylphenidate, atomoxetine does not increase extracellular dopamine levels in the striatum or nucleus accumbens [36]. Taking into account our previous work [21] demonstrating that dopaminergic neurons in the VTA are likely responsible for reanimation from general anesthesia, this suggests that the prefrontal cortex is not the primary anatomical target that mediates dopamine-mediated reanimation. More studies are needed to elucidate the specific dopaminergic circuits involved in reanimation from general anesthesia.

In studies of sleep neurobiology dating back for more than a half century, EEG changes have been used often to infer an arousal response in the absence of behavioral evidence. For example, pioneering work by Moruzzi and Magoun identified the reticular activating system by demonstrating that brainstem stimulation induced an awake EEG pattern in paralyzed cats [38]. The results of the present study suggest that a newly desynchronized EEG does not necessarily correlate with behavioral arousal, and stresses the importance of performing experiments that correlate changes in neurophysiology with changes in behavior. In our study, dextroamphetamine and atomoxetine both produced similar EEG changes, but only dextroamphetamine induced behavioral evidence of active emergence from general anesthesia.

The present results suggest that patients taking dextroamphetamine or methylphenidate may require higher doses of general anesthetics to maintain unconsciousness, and may be at increased risk of intraoperative awareness. This is supported by case reports suggesting that children with ADHD who take methylphenidate prior to surgery require higher doses of anesthetics [39, 40]. However, there are also reports suggesting that patients taking stimulants prior to surgery do not have increased anesthetic requirements, and therefore may be safely anesthetized without discontinuing the medications preoperatively [41]. Chambers et al. [42] found that BIS values during 1 MAC sevoflurane anesthesia were not significantly different in children taking stimulant medications vs. controls. However, since loss of consciousness occurs at approximately 1/3 of MAC for sevoflurane [43] it is possible that the dose of sevoflurane was too high to detect a difference between the two groups. Since ADHD is estimated to have a worldwide prevalence of 6 to 12% [44] and many children are now taking stimulant medications regularly, more studies are needed to establish how these medications affect anesthesia care.

The neural mechanisms underlying emergence from general anesthesia are not well understood, and treatment options are limited for common clinical problems such as delayed emergence, emergence delirium, and post-operative cognitive dysfunction. Although emergence from general anesthesia is currently treated as a passive process resulting from the pharmacokinetics of anesthetic drug clearance, agents that promote reanimation from general anesthesia may be clinically useful to accelerate recovery from general anesthesia, and treat common clinical problems related to emergence. The results of the present study suggest that dextroamphetamine may be clinically useful to promote emergence from general anesthesia in surgical patients, by stimulating dopamine release and activating D1 receptors in the brain. Conversely, selective inhibition of norepinephrine reuptake with atomoxetine does not induce reanimation from general anesthesia in rats, and therefore atomoxetine is unlikely to be clinically useful for facilitating emergence from general anesthesia in patients. Activating specific neural circuits that promote arousal may provide a novel approach to hasten recovery from general anesthesia after surgery, and may treat or obviate common clinical problems such as post-operative delirium and cognitive dysfunction.
